# Textural Optimization of Plant-Based Patties with Textured Fibrous Soy Protein and Konjac Glucomannan: A Response Surface Methodology Approach Targeting Springiness

**DOI:** 10.3390/foods15091503

**Published:** 2026-04-25

**Authors:** Hao Xu, Dongqin Liu, Weihua Du, Ke Hu, Jing Sun, Zhitong Xia, Zhengfei Yang, Yongqi Yin, Jiangyu Zhu

**Affiliations:** School of Food Science and Engineering, Yangzhou University, Yangzhou 225127, China; 243601119@stu.yzu.edu.cn (H.X.); 15150822975@163.com (D.L.); dwhlyl@163.com (W.D.); 243601204@stu.yzu.edu.cn (K.H.); 243601213@stu.yzu.edu.cn (J.S.); 243603219@stu.yzu.edu.cn (Z.X.); yzf@yzu.edu.cn (Z.Y.); yqyin@yzu.edu.cn (Y.Y.)

**Keywords:** plant-based meat analogues, textured fibrous soy protein, konjac glucomannan, protein–polysaccharide interactions, response surface methodology

## Abstract

Replicating the authentic masticatory properties of conventional animal meat remains a primary technical bottleneck for sustainable plant-based analogues. To address critical textural deficiencies like structural fragmentation, this study systematically optimized plant-based patty formulations. The independent and interactive effects of textured fibrous soy protein (TFSP), water, and konjac glucomannan (KGM) were quantified using single-factor experiments and Response Surface Methodology (RSM). Single-factor experiments revealed that springiness peaked at 60 g TFSP, 15 g water, and 10 g KGM, respectively, with excessive additions of each component resulting in structural network disruption. Designating springiness as the core metric, a reliable quadratic regression model identified the optimal matrix: 63.36 g TFSP, 14.39 g water, and 8.57 g KGM. Empirical validation achieved a maximum springiness of 1.56 mm and hardness of 5.51 N, with a negligible relative error (1.27%) from theoretical predictions. Mechanistically, KGM functioned as an active polymeric filler, interacting synergistically with hydrated protein fibers via hydrogen bonding and hydrophobic associations to reinforce the structural network. Comparative Texture Profile Analysis demonstrated that the optimized PBP exhibited a tender masticatory profile with hardness and springiness approximating conventional beef patties, while presenting lower chewiness and higher adhesiveness attributable to the water-binding capacity of KGM. Ultimately, this research provides mathematically validated engineering parameters and theoretical insights into protein–polysaccharide phase behaviors to facilitate the industrial manufacturing of premium plant-based meats.

## 1. Introduction

The global food system confronts unprecedented challenges precipitated by rapid population growth, climate change, and accelerating resource depletion. Transitioning toward sustainable dietary patterns has been internationally recognized as a fundamental strategy for mitigating these interconnected environmental and public health crises [1]. The World Health Organization has consistently recommended substantial reductions in processed and red meat consumption to alleviate the global burden of non-communicable diseases while promoting cardiovascular health [2]. Concurrently, the proliferation of plant-based dietary regimens aligns directly with the United Nations Sustainable Development Goals, particularly Goal 3 (Good Health and Well-being), Goal 12 (Responsible Consumption and Production), and Goal 13 (Climate Action) [3,4]. Consequently, developing high-quality plant-based food alternatives has emerged as an imperative interdisciplinary endeavor at the confluence of food science, nutritional engineering, and public health.

Plant-based meat analogues serve as prominent vectors for facilitating this dietary transition, offering sensory experiences approximating conventional meat while substantially mitigating greenhouse gas emissions and land utilization associated with livestock production [5]. Despite remarkable global market expansion, sustained consumer acceptance remains contingent upon high-fidelity replication of authentic meat characteristics, with textural attributes representing a critical determinant [6]. Structural parameters encompassing springiness, chewiness, cohesiveness, and hardness collectively dictate masticatory satisfaction and, by extension, repeated consumption. However, contemporary commercial plant-based patties frequently exhibit critical textural deficiencies. These products often present an insufficiently cohesive structural matrix, leading to undesirable fragmentation during thermal processing. Conversely, inappropriate biopolymer cross-linking may generate an excessively rigid and rubbery mouthfeel that deviates from expected sensory profiles [7]. Bridging this perceptual gap necessitates a systematic understanding of the physicochemical interactions governing structural components within the simulated meat matrix.

The structural foundation of plant-based meat analogues is predominantly constructed using extruded vegetable proteins processed under thermomechanical conditions that induce fibrillar organization [8]. However, previous investigations have demonstrated that reliance exclusively on textured proteins fails to yield a cohesive final product, as extruded fibrils alone cannot establish the continuous three-dimensional gel network necessary for structural integrity during mastication [9]. Accordingly, structural binders and plasticizing agents represent indispensable components for stabilizing these heterogeneous multiphase systems. Water functions as a critical plasticizer, facilitating molecular mobility, enabling component dispersion, and modulating viscoelastic properties. Furthermore, polysaccharide hydrocolloids have been frequently integrated to enhance binding capacity and modify rheological behavior. Konjac glucomannan (KGM), a neutral polysaccharide derived from Amorphophallus konjac, exhibits exceptional water-holding capacity and undergoes thermo-irreversible gelation upon alkaline deacetylation, rendering it particularly attractive for thermally processed food systems [10]. Prior research has documented that synergistic interactions—primarily mediated through hydrogen bonding and, where charge interactions permit, electrostatic associations—between soy proteins and KGM can substantially improve structural integrity and moisture retention [11]. Nevertheless, these investigations have predominantly focused on isolated components or examined binary interactions within simplified fluid systems. The intricate ternary interplay among textured fibrous soy protein (TFSP), water, and KGM within the highly sheared, semisolid environment characteristic of industrially processed meat batters has remained insufficiently characterized. This knowledge gap assumes particular significance given that mechanical history and compositional complexity may profoundly modulate the very interactions elucidated in simpler models, potentially yielding unpredictable textural outcomes during manufacturing scale-up.

The absence of systematic optimization regarding these ternary interactions presents a substantial impediment to rational formulation design. Establishing a mathematically validated, predictive framework is essential for achieving standardized, premium textural profiles across production batches. Response surface methodology (RSM) offers a robust statistical approach for evaluating multiple formulation variables and their complex interaction effects simultaneously, thereby enabling identification of precise compositional conditions required for structural optimization. Unlike conventional one-factor-at-a-time approaches, RSM efficiently captures nonlinear responses and synergistic or antagonistic relationships among components, rendering it particularly suitable for optimizing multicomponent food systems [12,13]. Therefore, this study aimed to systematically optimize the formulation of plant-based patties using a Box–Behnken response surface design, with springiness as the primary response variable. The independent and interactive effects of TFSP, water, and KGM on textural properties were quantified, and the underlying protein–polysaccharide interaction mechanisms are discussed to provide practical engineering parameters for industrial-scale production.

## 2. Materials and Methods

### 2.1. Materials and Chemicals

The primary protein ingredients utilized for formulating the plant-based patties (PBPs), specifically soy protein isolate (SPI, protein content ≥ 90%) and TFSP, were obtained from Huayi Biological Technology Co., Ltd. (Weifang, China). Textured wheat protein (TWP) was supplied by Lusu Biological Products Co., Ltd. (Linyi, China). Food-grade hydrocolloids, serving as structural binders, included KGM purchased from Qiangsen Konjac Technology Co., Ltd. (Wuhan, China) and methylcellulose (MC) provided by Tianhe Food Biological Products Co., Ltd. (Zhengzhou, China). Edible salt was sourced from Fengyuan Salt Industry Co., Ltd. (Weifang, China), and the composite seasoning mixture was acquired from Wang Shouyi Thirteen Spices Seasoning Group Co., Ltd. (Zhumadian, China). Commercial vegetable oil, serving as the lipid phase, was obtained from Yihai Kerry Arawana Holdings Co., Ltd. (Shanghai, China). Flavoring and coloring agents, including red yeast rice powder and garlic powder, were procured from Jiajie Natural Food Coloring Co., Ltd. (Guangzhou, China) and Weirui Food Co., Ltd. (Taizhou, China), respectively. All other chemicals and reagents used in this study were of analytical grade and obtained from Sinopharm Chemical Reagent Co., Ltd. (Shanghai, China).

### 2.2. Preparation of Plant-Based Patties

**Figure 1:**
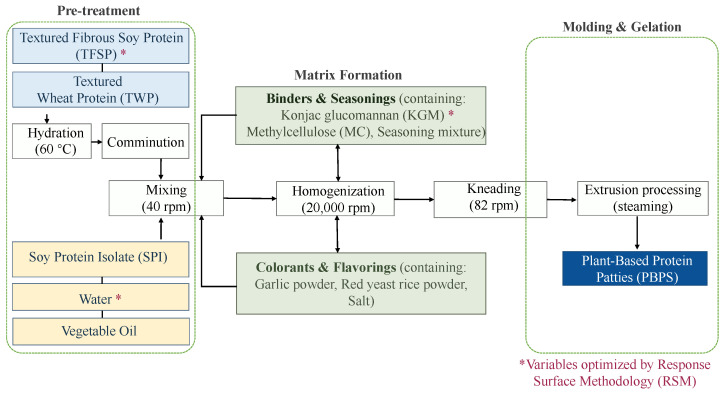
Flow chart for the preparation of plant-based patties.

The comprehensive preparation flowchart of the PBPs is illustrated in Figure 1. The fundamental formulation consisted of constant ingredients—TWP (15 g), SPI (6 g), MC (0.8 g), vegetable oil (10 g), seasoning mixture (0.7 g), garlic powder (0.4 g), red yeast rice powder (0.3 g), and edible salt (0.3 g)—alongside variable ingredients, specifically water (5–55 g), TFSP (15–90 g), and KGM (2.5–15 g), which were adjusted based on the experimental design.

The preparation process commenced with a hydration treatment. TFSP and TWP were individually soaked in a thermostatic water bath (HH-6, Changzhou Guohua Electric Appliance Co., Ltd., Changzhou, China) at 60 °C for 30–40 min. To guarantee uniform water absorption, the materials were gently agitated every 10 min until no hard cores remained within the fibrillar structures. Subsequently, a mechanical pressing method was applied to remove excess water, strictly controlling the final moisture content to 65–70% for TFSP and 35–50% for TWP. This precise moisture control is critical to preventing protein agglomeration and phase separation during subsequent mixing [14]. The hydrated TFSP and TWP were then transferred to a mechanical comminutor (FW100, Tianjin Taisite Instrument Co., Ltd., Tianjin, China) and shredded into distinct, meat-like protein fibers. Note that the weights of TFSP and TWP detailed in the formulation refer to their dry matter prior to hydration.

Concurrently, a stable oil-in-water (O/W) emulsion was fabricated at room temperature using a “stepwise dispersion-gradient stirring” strategy. SPI and the designated amount of water were initially mixed and stirred uni-directionally at 40 rpm for 30 s using a glass stirring rod to activate the surface-active sites of the protein. The vegetable oil was then incorporated into the aqueous protein phase in three sequential aliquots. Following each addition, the mixture was continuously stirred at 50 rpm for 20 s (total stirring duration of 1 min), yielding a homogenous, creamy O/W emulsion with no visible lipid phase separation [15].

For the batter formulation, the shredded TFSP and TWP, the pre-fabricated SPI emulsion, KGM, MC, and all requisite seasonings were comprehensively combined in a planetary mixer (E-1063, 1.3 kW, Shunran Co., Ltd., Shunde, China). An initial low-speed mixing phase (40 rpm for 2 min) was executed to facilitate preliminary dispersion of the components. The pre-mixed batter was then transferred to a meat grinder (JC-025, 0.3 kW, Chuzhijia Co., Ltd., Guangzhou, China) and homogenized at a high speed of 20,000 rpm for 5 min to form a refined, uniform “meat batter” matrix. The batter was returned to the planetary mixer and kneaded at a medium speed (82 rpm) for 10 min. This enhanced mechanical shearing promoted the cross-linking of protein molecules, thereby improving the water-holding capacity and springiness of the gel network [16].

**Figure 2:**
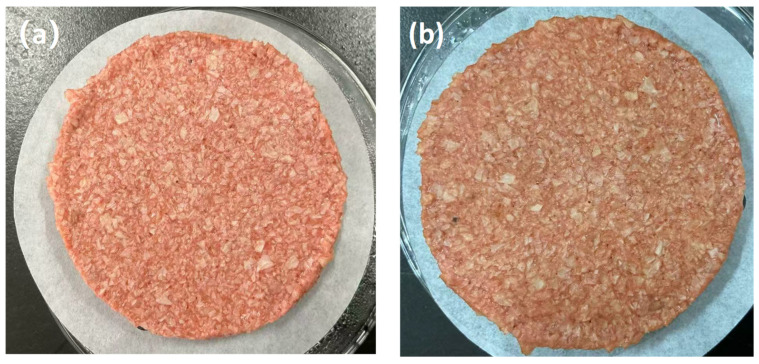
Representative photographs of plant-based patties (PBPs) at (**a**) the pre-cooked (molded) stage and (**b**) the post-cooked (steamed) stage.

Immediately following the mixing phase, the batter was extruded and molded using custom-made 304 stainless steel circular molds (50 mm in diameter, 10 mm in thickness) under uniform pressure. The formed PBPs were placed in Petri dishes and stored at 4 °C for subsequent thermal processing. The thermal gelation process was conducted using a multi-functional induction cooker (LV-220, 2200 W, Guangdong Peskoe Enterprises Group Co., Ltd., Zhanjiang, China). Distilled water was heated to boiling, and the heating power was then reduced to a simmering state (800–1000 W). The PBP samples were placed on food-grade silicone paper (compliant with Chinese National Standard GB4806.8) [17] over a stainless steel rack, ensuring a gap of 5–8 mm between adjacent samples and a vertical distance of ≥10 cm from the boiling water. Following a 5-min steaming duration, the cooked PBPs were rapidly removed and cooled to room temperature (25 °C). Representative photographs of the PBPs at the pre-cooked and post-cooked stages are presented in Figure 2.

### 2.3. Texture Profile Analysis

Textural properties of the cooked PBPs were evaluated via texture profile analysis (TPA) using a TA.XT.plus Texture Analyzer (Stable Micro Systems, Godalming, UK) equipped with a P50 cylindrical probe. Prior to instrumental analysis, the cooled samples were uniformly sectioned into distinct blocks (3 cm × 3 cm × 0.8 cm) and equilibrated at room temperature. The instrumental parameters for the double-compression test were configured as follows: pre-test speed of 2.0 mm/s, test speed of 1.0 mm/s, post-test speed of 2.0 mm/s, target compression distance of 50%, and a trigger force of 5 g. Five parallel replicates were measured for each sample formulation. The recorded TPA parameters encompassed hardness (N), springiness (mm), cohesiveness, chewiness (mJ), adhesiveness (mJ), and gumminess (N) [18].

### 2.4. Experimental Design and Optimization

To systematically optimize the structural formulation of the PBPs, a step-wise methodology incorporating single-factor experiments and RSM was deployed. Initially, single-factor trials were conducted by fixing the baseline parameters (TFSP at 60 g, water at 15 g, and KGM at 10 g) to eliminate confounding variables. The independent effects of the three core formulation variables—water addition (5, 15, 25, 35, 45, and 55 g), TFSP addition (15, 30, 45, 60, 75, and 90 g), and KGM addition (2.5, 5.0, 7.5, 10.0, 12.5, and 15.0 g)—on the overall textural profile of the PBPs were thoroughly evaluated.

Based on the empirical findings from the single-factor experiments, springiness (Y) was selected as the primary optimization response variable. We acknowledge that color and hardness are typically the primary consumer-perceived quality attributes in meat products. However, the specific focus on springiness in this study was deliberate and scientifically justified for the following reasons. First, color variation was minimized by design, as standardized colorants (red yeast rice powder) were included at fixed amounts across all experimental runs, meaning that color would not have been meaningfully discriminated across the BBD matrix and was therefore unsuitable as an optimization response. Second, hardness, while perceived early during mastication, exhibited monotonic declining trends with increasing TFSP and water additions in the single-factor experiments, rendering it less suitable for RSM optimization, which requires a clear non-linear response within the experimental domain. Third, springiness—defined as the ability of a deformed food to recover its original geometry after the compressive force is removed—reflects the elastic resilience of the gel network and is considered one of the most discriminating indicators of meat-like mouthfeel in plant-based analogues, as it captures both the elasticity of the biopolymer matrix and the cohesion of the interpenetrating network [8]. Fourth, and most critically, among all TPA parameters evaluated in the single-factor experiments, springiness exhibited the clearest non-linear (parabolic) response to all three formulation variables (TFSP, water, and KGM), with well-defined peak values within the tested ranges, making it uniquely suitable as the core response variable for RSM-based optimization. Subsequently, a three-factor, three-level Box–Behnken Design (BBD) was established to investigate the interaction effects of the key variables on the springiness of the PBPs [19]. The three selected independent variables were TFSP addition (Factor A), water addition (Factor B), and KGM addition (Factor C). The coded levels and actual experimental ranges of these variables are detailed in Table 1.

### 2.5. Statistical Analysis

All experimental determinations were executed in triplicate unless otherwise specified, and the analytical data were expressed as the mean ± standard deviation (SD). Data preprocessing and single-factor analysis of variance (ANOVA) were performed using SPSS software (Version 22.0, IBM Corp., Armonk, NY, USA), with significant differences determined utilizing Duncan’s multiple range test at a confidence level of *p* < 0.05. For the RSM optimization, the BBD experimental data were analyzed via Design-Expert software (Version 13.0.15, Stat-Ease Inc., Minneapolis, MN, USA). A second-order polynomial regression model was established to interpret the relationships between the response variable (springiness) and the independent factors. The statistical significance and adequacy of the predictive model were rigorously validated through ANOVA, focusing on the *F*-value, *p*-value, lack-of-fit statistic, and the coefficient of determination (*R*^2^).

## 3. Results and Discussion

### 3.1. Single-Factor Experimental Analysis

To systematically determine the optimal formulation parameters for the PBPs prior to response surface modeling, the independent effects of TFSP, water, and KGM on the composite textural properties were investigated. The textural profile, particularly springiness and cohesiveness, directly dictates the structural integrity and the simulation of authentic meat mouthfeel in plant-based alternatives.

#### 3.1.1. Effect of TFSP Addition

The influence of TFSP addition on the texture of the PBPs is summarized in Table 2. As the addition level increased from 15 g to 60 g, both springiness and cohesiveness exhibited an initial upward trend, reaching peak values of 1.56 mm and 0.28, respectively. Conversely, hardness decreased significantly (*p* < 0.05) from 22.80 N to 4.53 N across the tested range. Mechanistically, an addition of approximately 60 g provided an optimal ratio for the hydrated fibrillar proteins to interweave comprehensively with other matrix components, such as soy protein isolate and the polysaccharide hydrocolloid. This interweaving process is primarily driven by intermolecular forces, including hydrophobic associations and hydrogen bonding, which facilitate the filling of structural voids and the compaction of the microstructural network [20]. Consequently, the gel elasticity and internal binding capacity were maximized. When the addition level deviated from this optimal threshold, particularly exceeding 60 g, the cross-linking density of the protein molecules was observed to be either insufficient or excessively aggregated. Excessive addition likely disrupted the stability of the gel network due to the over-swelling of the protein fibers, thereby diminishing both springiness and cohesiveness.

Regarding the secondary TPA parameters, chewiness and gumminess both decreased monotonically as TFSP addition increased from 15 g to 90 g, declining from 4.00 mJ to 0.94 mJ and from 3.93 N to 1.07 N, respectively. This trend is consistent with the concomitant reduction in hardness, as chewiness and gumminess are mathematically derived from hardness and cohesiveness [21]. Adhesiveness also declined significantly with increasing TFSP (from 4.46 mJ to 1.48 mJ, *p* < 0.05), suggesting that higher protein fiber density reduces the surface stickiness of the matrix, likely due to reduced free water availability at the patty surface as the fibrillar network becomes more compact [22]. Considering the textural optimization results, a baseline of 60 g of TFSP was selected as the central point for subsequent response surface modeling.

#### 3.1.2. Effect of Water Addition

Water acts as a critical plasticizer and solvent within the composite gel system. As presented in Table 3, springiness and hardness varied significantly with water addition, where springiness peaked at an addition level of 15 g (1.51 mm). An appropriate hydration environment is essential to ensure the adequate dissolution and uniform dispersion of methylcellulose and KGM. This structural hydration facilitates the formation of a robust hydrocolloid gel driven by subsequent dehydration characteristics during thermal processing. A stable aqueous phase effectively prevents moisture migration and loss during cooking, thereby imparting a juicy texture analogous to real meat [23]. Furthermore, an optimal water level prevents the excessive swelling of textured proteins, thereby maintaining structural stability. Conversely, excessive water addition beyond 15 g resulted in a diluted matrix, leading to a loose structural network and a substantial decline in overall textural attributes. Notably, cohesiveness increased progressively with water content (from 0.19 at 5 g to 0.31 at 55 g), which may appear counterintuitive. This phenomenon can be attributed to the plasticizing effect of excess water on the biopolymer chains: while the overall network becomes mechanically weaker, the increased molecular mobility allows the matrix to deform more uniformly under compression without fracturing, yielding a higher ratio of the second compression force to the first and thus an apparent increase in cohesiveness [24]. This finding suggests that cohesiveness and springiness respond differently to hydration, and that springiness—rather than cohesiveness—is the more discriminating indicator of structural integrity in this system.

With respect to the secondary TPA parameters, chewiness, gumminess, and adhesiveness all decreased as water addition increased beyond 15 g (Table 3). Chewiness declined from 2.11 mJ at 5 g water to 0.74 mJ at 55 g, and gumminess followed a similar downward trend from 1.37 N to 0.83 N. This is mechanistically consistent with the progressive dilution of the gel network, which reduces the energy required for mastication [25]. Adhesiveness also decreased with excess water (from 4.40 mJ to 1.56 mJ), indicating that over-hydration reduces interfacial stickiness, possibly due to disruption of the hydrocolloid surface film formed by KGM and MC under optimal hydration conditions. Collectively, these results confirm that 15 g represents the optimal water addition level, balancing hydration of structural components with maintenance of network integrity across all TPA parameters.

#### 3.1.3. Effect of KGM Addition

The incorporation of KGM significantly influenced the structural network of the patties. As shown in Table 4, springiness and cohesiveness increased to maximum values of 1.53 mm and 0.30, respectively, at a dosage of 10 g before decreasing at higher concentrations. KGM is a neutral polysaccharide composed of β-1,4-linked D-glucose and D-mannose units. Upon thermal processing under the mildly alkaline conditions generated by the seasoning matrix, partial deacetylation of the acetyl substituents exposes hydrophobic domains along the KGM backbone. These exposed domains facilitate intermolecular aggregation through hydrophobic stacking and hydrogen bonding, producing a thermo-irreversible three-dimensional gel network that resists re-melting upon cooling [26]. Within the composite soy protein-KGM system, the high molecular weight and hydrodynamic volume of KGM generate a macromolecular crowding effect: KGM chains occupy a substantial portion of the aqueous phase, effectively increasing the local concentration of soy protein fibers in the continuous phase. This thermodynamic crowding drives accelerated protein-protein aggregation and cross-linking, resulting in a denser, more elastically resilient interpenetrating network. Consequently, springiness and cohesiveness increased markedly from 2.5 g to 10 g KGM, as the growing network density enhanced the elastic recovery capacity of the gel upon deformation. Beyond 10 g, however, excessive KGM concentration likely introduced phase separation effects, where over-concentrated polysaccharide chains competed with protein fibers for available water, disrupting the homogeneity of the network and reducing springiness. The superior water-holding capacity of KGM additionally retarded moisture loss during steaming, preserving gel plasticity and mitigating the thermal hardening that commonly occurs in protein-only systems [27].

The secondary TPA parameters followed distinct trends with increasing KGM. Chewiness and gumminess increased monotonically from 0.58 mJ and 0.77 N at 2.5 g KGM to 1.76 mJ and 1.60 N at 15 g, respectively, reflecting the progressive consolidation of the biopolymer gel network. Adhesiveness also increased substantially with KGM dosage (from 2.20 mJ to 4.54 mJ), which is directly attributable to the exceptional water-holding capacity of KGM that creates a viscous, adherent surface layer on the patty. This elevated adhesiveness may be considered a functional advantage in plant-based applications, as it promotes cohesion between the patty and coating materials during commercial processing. Therefore, 10 g was identified as the appropriate KGM inclusion level for the formulation, at which springiness and cohesiveness were maximized without the adverse phase separation effects associated with higher concentrations.

### 3.2. Optimization of Plant-Based Patties via Response Surface Methodology

#### 3.2.1. Predictive Model Building and ANOVA Analysis

Building upon the single-factor experimental results, a three-factor, three-level Box–Behnken design was executed to precisely optimize the formulation. Springiness was selected as the core response variable due to its direct correlation with consumer sensory acceptance and meat texture simulation. The experimental design matrix, consisting of 17 runs, and the corresponding measured elastic values are detailed in Table 5.

Prior to establishing the final predictive equation, the adequacy of various mathematical models—namely Linear, Two-Factor Interaction (2FI), Quadratic, and Cubic models—was systematically evaluated to determine the best fit for the experimental data. As detailed in Table 6, the quadratic model was unequivocally selected based on multiple rigorous statistical criteria. It exhibited a highly significant sequential *p*-value (<0.0001) and a non-significant lack-of-fit (*p* = 0.4102 > 0.05), indicating that this model accurately captures the data variation without substantial residual error. Furthermore, the quadratic model demonstrated the highest adjusted determination coefficient (Adjusted R^2^ = 0.9450) and predicted determination coefficient (Predicted R^2^ = 0.7963), alongside the lowest Predicted Residual Error Sum of Squares (PRESS = 0.4514). These parameters collectively confirm its superior predictive fidelity. Consequently, the experimental data were subjected to multiple regression analysis exclusively using the quadratic model, yielding the following second-order polynomial equation:

$$\begin{array}{c} Y=-13.313+0.313\times A+0.139\times B+0.925\times C-0.000\times AB\\ -0.008\;AC-0.003\;BC-0.002\;A^{2}-0.004\;B^{2}-0.021\;C^{2} \end{array}$$

The statistical significance of the chosen quadratic regression model was further validated through a detailed analysis of variance (ANOVA), as documented in Table 7. The established model exhibited a high F-value of 31.57 and a highly significant *p*-value (*p* < 0.0001), corroborating the robustness of the quadratic relationship. The lack-of-fit term remained non-significant, verifying that the experimental errors were well-controlled [28]. Furthermore, the coefficient of determination (*R*^2^) was calculated to be 0.9760, confirming an exceptional degree of correlation between the predicted and observed values. The linear terms of water (B) and KGM (C) exhibited significant effects (*p* < 0.05) on the response. Additionally, all quadratic terms (A^2^, B^2^, C^2^) presented highly significant negative effects (*p* < 0.0001 or *p* < 0.05), illustrating a non-linear parabolic trend typical of biopolymer complexation, which initially increases and subsequently decreases.

#### 3.2.2. Interaction Effects Among Formulation Variables

**Figure 3:**
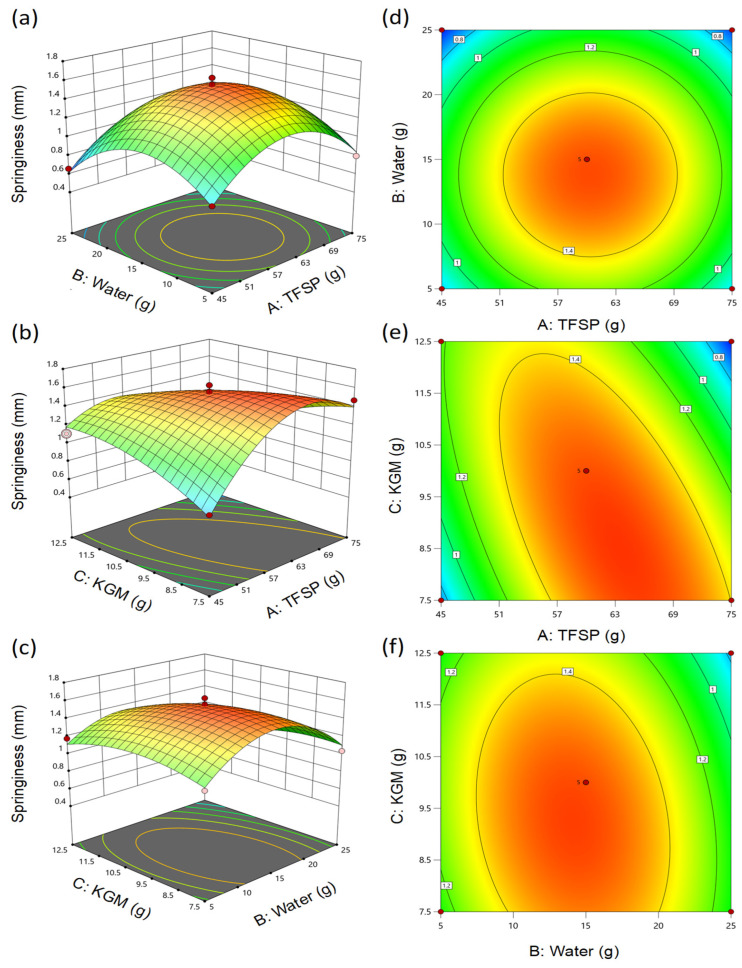
Response surface (3D, (**a**–**c**)) and contour plots (2D, (**d**–**f**)) illustrating the interactive effects of formulation variables on the springiness of plant-based patties: (**a**,**d**) interaction between TFSP and water; (**b**,**e**) interaction between TFSP and KGM; (**c**,**f**) interaction between water and KGM.

To elucidate the interaction effects among the independent variables, three-dimensional response surface plots and corresponding contour plots were generated (Figure 3). The geometrical shape of the contour plots quantifies the interaction intensity, where elliptical contours signify strong interactions. The surface plots displayed prominent convex topographies, further validating that optimal springiness occurs within the experimental boundaries.

Statistical analysis revealed a highly significant interaction between TFSP and KGM (AC, *p* = 0.0002). This profound synergistic effect is visually corroborated by the distinctly elliptical and diagonally tilted contour lines in Figure 3e. Conversely, the interaction between protein and water (AB, *p* = 1.0000) was negligible, which is graphically demonstrated by the contour ellipses in Figure 3d having principal axes perfectly parallel to the coordinate axes, indicating an independent phase behavior. Although the interaction between water and KGM was not statistically significant (BC, *p* = 0.1094), it was retained in the surface modeling to comprehensively illustrate the hydration competition within the matrix. The robust interaction between the protein and the polysaccharide is primarily attributed to their synergistic phase behavior driven by both hydrogen bonding and the excluded volume effect. While proteins form a cross-linked backbone during homogenization and thermal processing, the highly hydrophilic KGM molecules occupy a substantial hydrodynamic volume within the matrix. This spatial occupation effectively increases the local concentration of the soy proteins in the continuous aqueous phase, thermodynamically driving the protein fibers to aggregate and cross-link more densely. Consequently, KGM and TFSP construct a continuous, cohesive interpenetrating polymer network [29]. Conversely, the interaction between protein and water (AB, *p* = 1.0000) was negligible. This suggests that within the designated gradient, the ratio of protein to water operates somewhat independently, affecting the matrix hydration without fundamentally altering the specific cross-linking mechanisms dictated by the polysaccharide binder.

### 3.3. Validation of the Optimized Formulation

To validate the predictive model, three independent batches of plant-based patties were prepared using the optimized ingredient levels (63.36 g TFSP, 14.39 g water, and 8.57 g KGM) following the identical preparation protocol described in Section 2.2, including the same hydration, emulsification, mixing, kneading, molding, and steaming parameters. All other formulation components were held constant at the baseline levels specified in Section 2.2. Textural properties of the three validation batches were measured in five replicates per batch using the TPA protocol described in Section 2.3, and results are reported as mean ± standard deviation.

The fundamental objective of utilizing response surface methodology in this study was to identify the precise compositional ratio that maximizes the springiness of the plant-based patties, as this specific rheological parameter serves as a critical determinant of masticatory satisfaction and structural resilience. By mathematically solving the established second-order polynomial regression equation, the optimal formulation was derived. In the numerical optimization procedure conducted using Design-Expert, the goal for springiness was set to “maximize”, with the lower bound defined as the minimum observed experimental value (0.59 mm) and the upper bound as the maximum observed value (1.63 mm). The “maximize” criterion was selected—rather than a specific target value—because the primary objective of this study was to identify the formulation that achieves the highest attainable springiness within the tested compositional range, consistent with the goal of maximizing meat-like elastic recovery. The desirability function value for the identified optimal formulation (63.36 g TFSP, 14.39 g water, 8.57 g KGM) was 0.952, indicating a high degree of optimization confidence and confirming that this solution closely approaches the theoretical maximum springiness within the defined experimental space.

The empirical springiness value was determined to be 1.56 ± 0.05 mm (Table 8). A comparative analysis between the theoretical prediction (1.58 mm) and the experimental outcome (1.56 mm) reveals a remarkable concordance, yielding a negligible relative error of approximately 1.27%. This exceptionally high degree of fitting effectively verifies that no significant overfitting occurred during the statistical modeling process. Furthermore, it substantiates the high precision and adequacy of the Box–Behnken design in capturing the complex, non-linear ternary interactions among the protein fibrous backbone, the aqueous plasticizer, and the polysaccharide binder. Consequently, the validated regression model is confirmed to be highly robust, providing standardized engineering parameters and a reliable theoretical framework for the industrial scale-up manufacturing of premium vegetarian meat products with targeted textural profiles.

**Table 8:** Predicted and experimental values of springiness under optimal process conditions.

Item	Springiness (mm)
Experimental value	1.56 ± 0.05
Predicted value	1.58

### 3.4. Comparison of the Optimized Plant-Based Patties with Conventional Animal Meat Patties

To comprehensively evaluate the textural fidelity of the optimized plant-based patties (PBPs), a comparative analysis was conducted against conventional animal meat patties (beef, chicken, and lamb) using Texture Profile Analysis (TPA) parameters sourced from recent literature. Table 9 presents the experimental TPA values of the optimized PBP alongside the literature-derived values for conventional beef [30], chicken [31], and lamb patties [32]. As noted by Bohrer [33], achieving the precise textural characteristics of traditional meat analogues remains a formidable challenge due to the distinct physicochemical behavior of plant proteins compared to myofibrillar proteins during thermal gelation.

It is important to note that while springiness was designated as the primary optimization response variable in this study, the comparative analysis presented in this section encompasses the full TPA profile of the optimized PBP. This approach is intentional and scientifically justified: RSM optimization was performed targeting springiness because it was identified as the most sensitive and discriminating parameter for formulation development, exhibiting the clearest non-linear response to all three formulation variables within the experimental domain. However, a comprehensive evaluation of the optimized product against conventional meat benchmarks necessarily involves all measurable textural attributes, as consumers and industry evaluators assess meat quality holistically rather than on a single parameter. Regarding springiness specifically, the optimized PBP achieved a value of 1.56 ± 0.05 mm, which falls within the range reported for conventional beef patties in the literature (typically 0.80–1.80 mm depending on fat content and cooking method), indicating that the optimized formulation successfully replicates the elastic recovery characteristics of conventional meat to a satisfactory degree. However, we acknowledge that direct numerical comparison of springiness with the three reference studies cited in Table 9 was not possible, as these studies did not report springiness values under comparable measurement conditions. The remaining TPA parameters are therefore discussed below to provide a complete textural profile and contextual understanding of how the optimized PBP performs relative to conventional meat benchmarks.

The optimized PBP exhibited a hardness of 5.51 ± 0.03 N, which is generally lower than the conventional beef (9.97 N) [30] and lamb patties (16.41 N) [32], and significantly softer than typical commercial chicken patties (54.58 N) [31]. This indicates that while the KGM and TFSP successfully formed a cohesive interpenetrating gel, the resulting matrix presents a more tender masticatory profile compared to the dense actomyosin networks in real meat. The cohesiveness of the PBP (0.23 ± 0.02) was slightly lower than that of the animal meat counterparts (typically ranging from 0.35 to 0.45). This variation is characteristic of the fibrous but less highly cross-linked nature of textured soy matrices, which lack the continuous myofibrillar architecture responsible for the high structural cohesion observed in genuine muscle tissue.

Furthermore, the chewiness (1.98 ± 0.02 mJ) and gumminess (1.27 ± 0.15 N) of the PBP were correspondingly lower than the conventional meat references. Since these secondary TPA parameters are mathematically derived from hardness and cohesiveness, the tender nature of the PBP directly translates to a reduced energy requirement for mastication. Interestingly, the adhesiveness of the PBP (3.25 ± 0.04 mJ) was notably higher than that of the reference animal meat models (e.g., chicken at 0.16 mJ). This unique attribute can be directly ascribed to the strong water-binding capacity and viscous gelation properties of the KGM binder acting within the interstitial spaces of the soy protein network. Overall, the optimized formulation provides a highly acceptable, tender, and cohesive structural matrix that effectively simulates the sensory bite of conventional meat patties, whilst offering a customizable structural platform for targeted textural modifications.

It is important to acknowledge that the relatively low hardness (5.51 N) and cohesiveness (0.23) of the optimized PBP, compared to conventional meat patties, may raise practical concerns regarding shape retention and structural stability during cooking and handling. In theory, reduced cohesiveness increases the susceptibility of the matrix to fragmentation under mechanical stress, particularly during pan-frying or grilling where thermal shrinkage forces are considerable. However, in the present study, the thermo-irreversible gelation of KGM plays a critical compensatory role: upon heating, KGM undergoes irreversible network consolidation via deacetylation-driven hydrogen bonding, which effectively locks the interpenetrating biopolymer architecture and mitigates deformation [26]. Furthermore, the inclusion of MC as a co-binder provides additional thermal gelation capacity, as MC exhibits a reverse thermal gelation behavior that increases gel strength precisely during the cooking phase, thereby maintaining patty integrity under heat [20]. The molding geometry (50 mm diameter, 10 mm thickness) and the controlled steaming protocol (5 min at 800–1000 W) further support dimensional stability by avoiding the mechanical agitation associated with direct-contact cooking methods.

Nonetheless, we acknowledge that long-term cold storage stability and the effects of repeated freeze–thaw cycles on cohesiveness warrant systematic investigation in future work. Reformulation strategies such as increasing MC concentration, incorporating transglutaminase as a protein cross-linking agent, or introducing structured plant fiber networks could be explored to further improve shape fidelity and handling robustness for commercial applications.

## 4. Conclusions

In this study, the structural architecture of plant-based patties was systematically optimized using response surface methodology. The predictive model identified an optimal matrix consisting of 63.36 g of TFSP, 14.39 g of water, and 8.57 g of KGM, which achieved a maximum springiness of 1.56 mm. The empirical validation demonstrated excellent agreement with the theoretical prediction, thereby confirming the model’s reliability. Mechanistically, KGM functioned as a critical structural modulator by engaging in synergistic interactions with the proteinaceous fibrous network, primarily through hydrogen bonding and hydrophobic interactions, thereby facilitating the formation of a cohesive biopolymeric architecture. This intermolecular cross-linking not only mitigated the common structural defects observed in plant-based analogues but also conferred an optimal hardness of 5.51 N, effectively replicating the textural attributes of conventional meat products during oral processing. Notably, while these findings represent a significant advancement in textural mimicry, several limitations warrant attention in future work: the effects of TFSP and KGM on color attributes, cooking loss, and microstructure were not evaluated within the present RSM framework, and the absence of sensory panel evaluation limits direct consumer acceptance validation. Future investigations incorporating these parameters, alongside volatile compound profiling and dynamic in vitro gastrointestinal digestion models, will be essential for the rational design of next-generation sustainable meat alternatives with enhanced consumer acceptability.

## Figures and Tables

**Table 1:** Variables and their coded levels in the Box–Behnken design for optimizing PBP formulations.

Levels	Factor A: TFSP (g)	Factor B: Water (g)	Factor C: KGM (g)
−1	45	5	7.5
0	60	15	10
1	75	25	12.5

**Table 2:** Effect of TFSP addition on the texture of plant-based patties.

TFSP (g)	Hardness (N)	Springiness (mm)	Cohesiveness	Chewiness (mJ)	Adhesiveness (mJ)	Gumminess (N)
15	22.80 ± 2.89 ^a^	1.02 ± 0.05 ^b^	0.16 ± 0.01 ^c^	4.00 ± 0.68 ^a^	4.46 ± 0.09 ^a^	3.93 ± 1.19 ^a^
30	12.33 ± 1.07 ^b^	1.11 ± 0.02 ^ab^	0.17 ± 0.03 ^c^	2.29 ± 0.08 ^b^	3.87 ± 0.66 ^a^	2.03 ± 0.15 ^b^
45	6.97 ± 0.95 ^c^	1.12 ± 0.11 ^ab^	0.17 ± 0.01 ^c^	1.55 ± 0.14 ^b^	3.65 ± 0.15 ^a^	1.53 ± 0.12 ^b^
60	5.43 ± 0.21 ^c^	1.56 ± 0.10 ^a^	0.28 ± 0.02 ^a^	1.54 ± 0.08 ^b^	3.43 ± 0.35 ^ab^	1.37 ± 0.06 ^b^
75	4.57 ± 0.86 ^c^	0.85 ± 0.10 ^b^	0.27 ± 0.01 ^a^	0.95 ± 0.32 ^b^	2.55 ± 0.31 ^bc^	1.17 ± 0.15 ^b^
90	4.53 ± 0.47 ^c^	0.81 ± 0.06 ^b^	0.21 ± 0.01 ^b^	0.94 ± 0.06 ^b^	1.48 ± 0.28 ^c^	1.07 ± 0.25 ^b^

Note: Different lowercase letters within the same column indicate significant differences (*p* < 0.05).

**Table 3:** Effect of water addition on the texture of plant-based patties.

Water (g)	Hardness (N)	Springiness (mm)	Cohesiveness	Chewiness (mJ)	Adhesiveness (mJ)	Gumminess (N)
5	4.27 ± 0.42 ^b^	0.85 ± 0.21 ^b^	0.19 ± 0.01 ^c^	2.11 ± 0.89 ^a^	4.40 ± 0.97 ^a^	1.37 ± 0.29 ^a^
15	5.57 ± 0.06 ^a^	1.51 ± 0.33 ^a^	0.20 ± 0.03 ^c^	1.53 ± 0.12 ^ab^	3.56 ± 0.05 ^ab^	1.03 ± 0.12 ^b^
25	4.47 ± 0.47 ^b^	0.96 ± 0.12 ^b^	0.20 ± 0.03 ^bc^	0.84 ± 0.17 ^b^	2.50 ± 0.53 ^bc^	0.90 ± 0.10 ^b^
35	4.10 ± 0.30 ^bc^	0.87 ± 0.09 ^b^	0.23 ± 0.03 ^bc^	0.79 ± 0.11 ^b^	2.06 ± 0.24 ^c^	0.87 ± 0.12 ^b^
45	3.83 ± 0.38 ^bc^	0.86 ± 0.10 ^b^	0.25 ± 0.01 ^b^	0.76 ± 0.31 ^b^	1.88 ± 0.53 ^c^	0.87 ± 0.15 ^b^
55	3.50 ± 0.17 ^c^	0.81 ± 0.22 ^b^	0.31 ± 0.04 ^a^	0.74 ± 0.29 ^b^	1.56 ± 0.74 ^c^	0.83 ± 0.06 ^b^

Note: Different lowercase letters within the same column indicate significant differences (*p* < 0.05).

**Table 4:** Effect of KGM addition on the texture of plant-based patties.

KGM (g)	Hardness (N)	Springiness (mm)	Cohesiveness	Chewiness (mJ)	Adhesiveness (mJ)	Gumminess (N)
2.50	3.93 ± 0.12 ^c^	0.67 ± 0.13 ^c^	0.15 ± 0.01 ^d^	0.58 ± 0.05 ^c^	2.20 ± 0.36 ^d^	0.77 ± 0.06 ^b^
5.00	4.27 ± 0.29 ^c^	0.74 ± 0.12 ^c^	0.16 ± 0.01 ^cd^	0.58 ± 0.09 ^c^	2.64 ± 0.46 ^cd^	0.77 ± 0.12 ^b^
7.50	4.77 ± 0.40 ^bc^	0.77 ± 0.06 ^c^	0.18 ± 0.02 ^bcd^	0.68 ± 0.14 ^c^	2.67 ± 0.47 ^cd^	0.87 ± 0.15 ^b^
10.00	5.70 ± 0.87 ^ab^	1.53 ± 0.14 ^a^	0.30 ± 0.07 ^a^	1.51 ± 0.19 ^b^	3.33 ± 0.30 ^bc^	1.33 ± 0.21 ^a^
12.50	5.93 ± 1.22 ^ab^	1.25 ± 0.11 ^b^	0.24 ± 0.03 ^ab^	1.69 ± 0.03 ^ab^	3.66 ± 0.56 ^ab^	1.50 ± 0.17 ^a^
15.00	6.87 ± 0.40 ^a^	1.24 ± 0.04 ^b^	0.22 ± 0.03 ^bc^	1.76 ± 0.03 ^a^	4.54 ± 0.62 ^a^	1.60 ± 0.26 ^a^

Note: Different lowercase letters within the same column indicate significant differences (*p* < 0.05).

**Table 5:** Measured elastic values (springiness) of each experimental group in the Box–Behnken design.

Run	Factor A (g)	Factor B (g)	Factor C (g)	Springiness (mm)
1	45.00	5.00	10.00	0.81
2	75.00	5.00	10.00	0.80
3	45.00	25.00	10.00	0.66
4	75.00	25.00	10.00	0.65
5	45.00	15.00	7.50	0.77
6	75.00	15.00	7.50	1.47
7	45.00	15.00	12.50	1.11
8	75.00	15.00	12.50	0.59
9	60.00	5.00	7.50	1.10
10	60.00	25.00	7.50	1.04
11	60.00	5.00	12.50	1.18
12	60.00	25.00	12.50	0.80
13	60.00	15.00	10.00	1.48
14	60.00	15.00	10.00	1.45
15	60.00	15.00	10.00	1.63
16	60.00	15.00	10.00	1.56
17	60.00	15.00	10.00	1.63

Note: Factors A, B, and C represent the actual addition levels of TFSP, water, and KGM, respectively.

**Table 6:** Fit summary statistics for evaluating the adequacy of various response surface models.

Source	Sum of Squares	df	Mean Square	F-Value	*p*-Value
	**Sequential model sum of squares**				
Mean vs. Total	20.64	1	20.64		
Linear vs. Mean	0.1329	3	0.0443	0.2764	0.8414
2FI vs. Linear	0.3977	3	0.1326	0.7864	0.5284
Quadratic vs. 2FI	1.63	3	0.5442	71.47	<0.0001
Cubic vs. Quadratic	0.0255	3	0.0085	1.22	0.4102
Residual	0.0278	4	0.0070	/	/
Total	22.85	17	1.34	/	/
	**Lack of fit tests**				
Linear	2.06	9	0.2284	32.87	0.0021
2FI	1.66	6	0.2763	39.76	0.0016
Quadratic	0.0255	3	0.0085	1.22	0.4102
Cubic	0.0000	0	/	/	/
Pure Error	0.0278	4	0.0070	/	/
	**Model summary statistics**				
	**Std. Dev.**	**R^2^**	**Adjusted R^2^**	**Predicted R^2^**	**PRESS**
Linear	0.4003	0.0600	−0.1570	−0.5186	3.37
2FI	0.4106	0.2394	−0.2170	−1.0406	4.52
Quadratic	0.0873	0.9760	0.9450	0.7963	0.4514
Cubic	0.0834	0.9875	0.9498	/	/

**Table 7:** Model statistical analysis data (ANOVA) for the response surface quadratic model.

Source	Sum of Squares	*df*	Mean Square	*F*-Value	*p*-Value	Significance
Model	2.16	9	0.2403	31.57	<0.0001	**
A	0.0032	1	0.0032	0.4203	0.5375	ns
B	0.0684	1	0.0684	8.99	0.0200	*
C	0.0612	1	0.0612	8.04	0.0252	*
AB	0.0000	1	0.0000	0.0000	1.0000	ns
AC	0.3721	1	0.3721	48.87	0.0002	**
BC	0.0256	1	0.0256	3.36	0.1094	ns
A^2^	0.7876	1	0.7876	103.44	<0.0001	**
B^2^	0.6322	1	0.6322	83.03	<0.0001	**
C^2^	0.0739	1	0.0739	9.71	0.0169	*
Residual	0.0533	7	0.0076			
Lack of Fit	0.0255	3	0.0085	1.22	0.4102	ns
Pure Error	0.0278	4	0.0070			
Cor Total	2.22	16				

Note: ** indicates high significance (*p* < 0.01); * indicates significance (*p* < 0.05); ns indicates not significant.

**Table 9:** Texture characteristics of plant-based meat patties under optimal process conditions compared with conventional animal meat patties.

Item	Hardness (N)	Cohesiveness	Chewiness (mJ)	Adhesiveness (mJ)	Gumminess (N)
Experimental value	5.51 ± 0.03	0.23 ± 0.02	1.98 ± 0.02	3.25 ± 0.04	1.27 ± 0.15
Beef patties	9.97 ± 0.85	0.35 ± 0.02	5.07 ± 0.35	1.25 ± 0.10	3.49 ± 0.20
Chicken patties	54.58 ± 1.20	0.45 ± 0.03	12.50 ± 0.80	0.16 ± 0.19	24.56 ± 1.10
Lamb patties	16.41 ± 1.05	0.38 ± 0.04	6.20 ± 0.55	0.85 ± 0.15	6.23 ± 0.45

Note: Values for beef, chicken, and lamb patties represent baseline control data (100% meat without structural binders) adapted respectively from Serdaroğlu et al. [30], Kesemen & Akköse [31], and Guedes-Oliveira et al. [32], providing a representative framework for conventional meat texture simulation. We acknowledge that this comparison is limited to three conventional meat patty studies, primarily due to the significant heterogeneity in TPA measurement protocols (probe geometry, compression ratio, sample dimensions) across the published literature, which precludes broader direct numerical comparisons.

## Data Availability

The data presented in this study are available on request from the corresponding author due to the data are not publicly available due to privacy or ethical restrictions.
